# Recent applications of click chemistry for the functionalization of gold nanoparticles and their conversion to glyco-gold nanoparticles

**DOI:** 10.3762/bjoc.14.2

**Published:** 2018-01-03

**Authors:** Vivek Poonthiyil, Thisbe K Lindhorst, Vladimir B Golovko, Antony J Fairbanks

**Affiliations:** 1 Otto Diels Institute of Organic Chemistry, Christiana Albertina University of Kiel, Otto-Hahn-Platz 3/4, Kiel, 24098, Germany; 2 Department of Chemistry, University of Canterbury, Private Bag 4800, Christchurch, 8140, New Zealand; 3 The MacDiarmid Institute for Advanced Materials and Nanotechnology, Wellington, 6140, New Zealand; 4 Biomolecular Interaction Centre, University of Canterbury, Private Bag 4800, Christchurch 8140, New Zealand

**Keywords:** azide–alkyne Huisgen cycloaddition, carbohydrates, click chemistry, glyco-gold nanoparticles, triazole

## Abstract

Glycoscience, despite its myriad of challenges, promises to unravel the causes of, potential new detection methods for, and novel therapeutic strategies against, many disease states. In the last two decades, glyco-gold nanoparticles have emerged as one of several potential new tools for glycoscientists. Glyco-gold nanoparticles consist of the unique structural combination of a gold nanoparticle core and an outer-shell comprising multivalent presentation of carbohydrates. The combination of the distinctive physicochemical properties of the gold core and the biological function/activity of the carbohydrates makes glyco-gold nanoparticles a valuable tool in glycoscience. In this review we present recent advances made in the use of one type of click chemistry, namely the azide–alkyne Huisgen cycloaddition, for the functionalization of gold nanoparticles and their conversion to glyco-gold nanoparticles.

## Introduction

Metal nanoparticles (NPs), with their unique physicochemical properties, have drawn significant interest in recent years, and are expected to form the basis of many biological and technological innovations during the remainder of the 21st century [1]. Gold nanoparticles (AuNPs) are one of the most significant and stable classes of metal NPs [2] and have potential applications in optics [3], biology [4] and catalysis [5].

Carbohydrates are one of the classes of molecules that are essential for life. Although they are involved in many important biological processes, it is now well established that the binding interactions of a particular oligosaccharide, either with another carbohydrate or more commonly with carbohydrate-binding proteins (lectins), are generally weak. In order to augment these low affinity interactions, oligosaccharides usually bind lectins in a multivalent cooperative fashion. This avidity is significantly greater than the sum of the individual monomeric carbohydrate–protein interactions, and is sometimes referred to as the ‘cluster glycoside’ effect [6]. In order to study biological processes that involve these types of carbohydrate–protein interactions, it is therefore essential to present carbohydrates in a multivalent fashion. For that purpose, different scaffolds, such as peptides, proteins, lipids, and synthetic polymers, have all been used [7].

The search for better scaffolds for the presentation of multivalent carbohydrate structures led to the development of self-assembled monolayers (SAMs) of carbohydrates on the spherical surface of AuNPs. In 2001, the Penadés group reported the first synthesis of AuNPs with attached carbohydrates [8]. These systems, termed ‘glyco-gold nanoparticles’ (GAuNPs), were comprised of AuNPs with the surface Au atoms covalently attached to thiols of thiol-terminated oligosaccharides [8]. It was found that GAuNPs could be used as mimics of the glycocalyx to study both carbohydrate–carbohydrate and carbohydrate–protein interactions [9–10]. Other applications of GAuNPs, as sensors for various biomolecules and toxins, including the detection of pathogenic agents such as viruses and bacteria, have also been reported by various groups [11–16].

Since the first report by Penadés [8], numerous methods have been developed for the synthesis of GAuNPs. However, recent use of click chemistry for the functionalization of AuNPs and their conversion to GAuNPs has increased significantly. This short review, after giving a brief introduction to general methods for GAuNP synthesis, will focus on both potential advantages and issues of using click chemistry for the functionalization of AuNPs and their conversion to GAuNPs.

## Review

### Methods for the synthesis of GAuNPs

**Figure 1:**
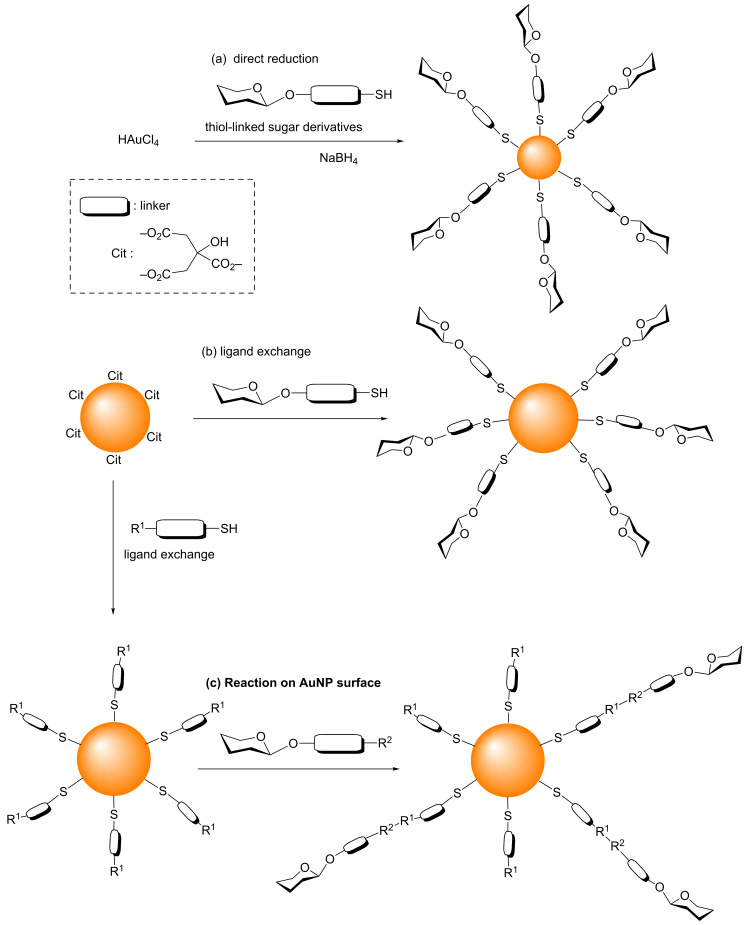
The three major methods for the synthesis of GAuNPs. (a) Direct reduction of an Au^3+^ salt in the presence of thiol-linked sugar derivatives to obtain GAuNPs of sizes smaller than 10 nm. (b) Exchange of citrate molecules (cit) on citrate-stabilized AuNPs with thiol-linked sugar derivatives to obtain GAuNPs of various sizes. (c) Reactions of AuNPs (obtained after ligand exchange) with suitably functionalized sugar derivatives.

In general, there are three main methods that can be used to synthesize GAuNPs (Figure 1). The first one is a direct method, involving the reduction of HAuCl_4_ in the presence of carbohydrate derivatives with a thiol end group, which is generally attached to the reducing terminus by a linker (Figure 1a) [8,14,17–27].

The second method is a ligand exchange reaction involving the replacement of the ligands on pre-formed AuNPs with thiol-linked carbohydrate derivatives (Figure 1b). The most frequently employed approach here is to first synthesize citrate-stabilized AuNPs (Cit-AuNPs) [28], and then to replace the citrate ligands with the desired thiol-linked carbohydrate derivatives [29–30]. Ligand exchange on the AuNP surface is driven by the higher binding affinity of Au for the thiol than for citrate, due to the significant energy difference between Au–S (≈40 kcal·mol^−1^) and Au–O_COOH_ (≈2 kcal·mol^−1^) interactions [31].

The third method involves the chemical reaction of functional groups of ligands attached to the surface of pre-formed AuNPs with suitably functionalized carbohydrates (Figure 1c). Various types of reaction, such as reductive amination [32], oxime formation [33], amidation [34], and perfluorophenyl azide (PFPA) photocoupling [35–36], have been used to functionalize the surface of AuNPs with carbohydrates. The detailed information regarding the synthesis and application of GAuNPs can be found in the reviews by Penadés and co-workers [9,26] and also in a recent review by Compostella et al. [10]. In this regard, azide–alkyne click chemistry is an attractive approach that could be used to synthesize GAuNPs.

### The functionalization of AuNPs using the azide–alkyne Huisgen cycloaddition

#### AuNP surface modification using NCAAC

**Scheme 1:**
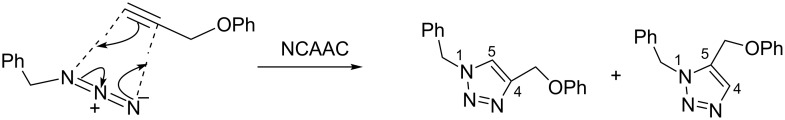
The non-catalysed azide–alkyne Huisgen cycloaddition (NCAAC) between an organic azide (1,3-dipole) and an alkyne (dipolarophile) resulting in the formation of regioisomeric triazole products.

The azide–alkyne Huisgen cycloaddition (AAC) is a 1,3-dipolar cycloaddition between an organic azide and an alkyne that gives triazole products [37–38]. The non-catalysed azide–alkyne Huisgen cycloaddition (NCAAC) is very slow, and gives a mixture of 1,4- and 1,5-triazole regioisomers (Scheme 1) [39]. Interest in and applications of the AAC have surged over the past 15 or so years, since the introduction of Cu(I) catalysis, which led to significant improvements in both the regioselectivity and rates of the reaction [40–41]. The versatility of the Cu(I)-catalysed azide–alkyne Huisgen cycloaddition (CuAAC) has been demonstrated by its robustness, insensitivity to water and oxygen, and its applicability to a wide range of substrates [42–44]. Although the AAC has been used by many groups to modify the surface of AuNPs [45–48], until recently it has only rarely been used to synthesize GAuNPs.

**Scheme 2:**
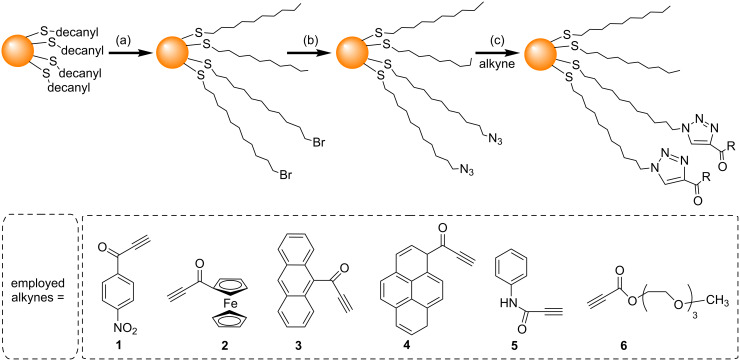
Ligand exchange and NCAAC on an AuNP surface. Reagents and conditions: (a) Br(CH_2_)_11_SH in DCM, 60 h, rt; (b) NaN_3_, DCM/DMSO, 48 h; (c) R = propyn-1-one derivatives, 24–96 h in dioxane, or 1:1 hexane/dioxane [45].

In 2006, Fleming et al. used the NCAAC to attach a series of different species to AuNPs [45]. Small AuNPs (1.8 nm) were used as the substrates for the NCAAC because of their ease of synthesis, high solubility, and good ligand exchange properties. A two-phase Brust–Schiffrin method (BSM) [49] was first used to synthesize decanethiol-stabilized AuNPs. These particles were then reacted with 11-bromo-1-undecanethiol to replace some of the decanethiol ligands with Br-terminated undecanethiol ligands (Scheme 2). Nucleophilic substitution by reaction with NaN_3_ then resulted in AuNPs with mixed monolayers containing 52% N_3_- and 44% CH_3_-terminated alkanethiol ligands. A series of alkynes were synthesised, including derivatives of nitrobenzene (**1**), ferrocene (**2**), anthracene (**3**), pyrene (**4**), aniline (**5**), and polyethylene glycol (**6**) all of which contained a carbonyl group next to the alkyne to increase the rate of triazole formation [50]. NCAAC between the azide-decorated AuNPs and the alkyne derivatives (**1**–**6**) was then performed (Scheme 2). Although a small amount of the AuNPs underwent irreversible aggregation, the majority of the AuNPs (>90%) remained soluble, and could be separated from aggregates after the reaction. Although Fleming et al. successfully performed NCAAC on these AuNPs, the yields (i.e., the extent of the azide conversion to triazole) were low (22%, or 54% in one specific case) even after 60 hours [45,51].

**Scheme 3:**
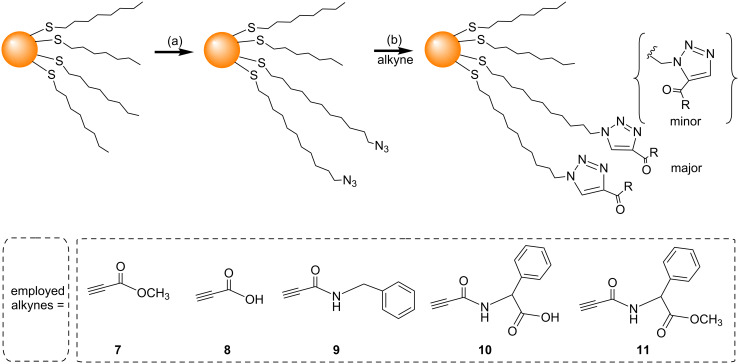
Azide functionalization and NCAAC on an AuNP surface using electron deficient alkynes. Reagents and conditions: (a) HS(CH_2_)_11_N_3_, C_6_H_6_, rt, 7 h; (b) THF, rt, 16 h [52].

**Scheme 4:**
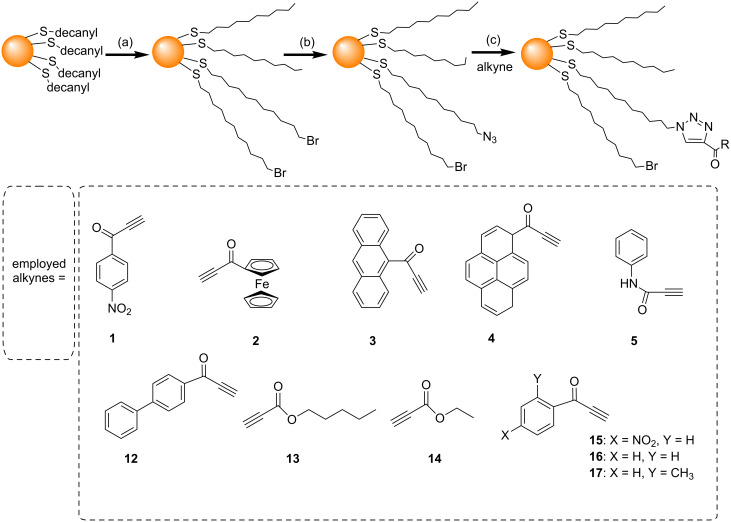
NCAAC performed under hyperbaric conditions. Reagents and conditions: (a) Br(CH_2_)_11_SH in C_6_H_6_, 48 h, rt; (b) NaN_3_ in C_6_H_6_/DMSO, 48 h; (c) R = propyn-1-one derivatives, DCM, 11000 atm, 25 °C, 15–24 h [48].

Following the work of Fleming et al*.,* several groups have investigated the use of different conditions to try and increase the efficiency of the NCAAC on the surface of AuNPs. Limapichat et al. used other electron deficient alkynes (**7**–**11**) as substrates for the NCAAC, and observed that 75% of the azides on the AuNP surface underwent cycloaddition in 16 hours (Scheme 3) [52]. Ismaili et al. carried out the NCAAC with a number of terminal-acyl alkynes (**1**–**5** and **12**–**17**) under hyperbaric conditions (11000 atm pressure), and observed 80% or higher conversions within 15 to 24 hours (Scheme 4) [48].

#### AuNP surface modification using strain-promoted azide–alkyne cycloaddition

**Scheme 5:**
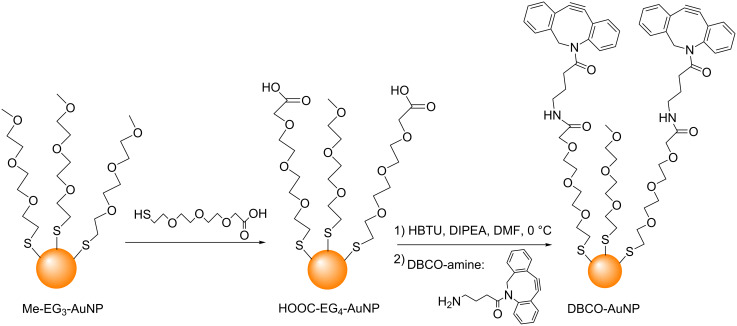
The synthesis of AuNPs functionalized with strained alkyne derivatives. HBTU = *O*-benzotriazole-*N*,*N*,*N*',*N*'-tetramethyluroniumhexafluorophosphate; DIPEA = *N,N*-diisopropylethylamine [57].

**Scheme 6:**
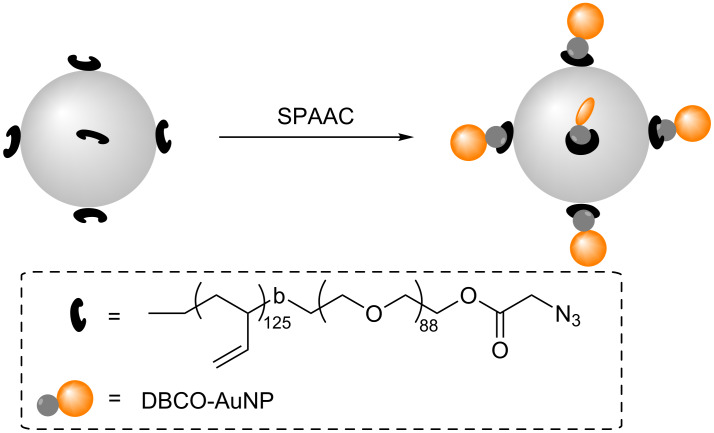
A schematic representation of the SPAAC between azide-functionalized polymersomes and strained alkyne-functionalized AuNPs (DBCO-AuNPs) in water [57].

In 2014, Workentin and co-workers used the strain promoted azide–alkyne cycloaddition (SPAAC) [53–56] to modify AuNP surfaces [57]. Firstly 2.8 nm AuNPs functionalized with strained dibenzocyclooctyne derivatives (DBCO-AuNPs) were synthesized in two steps (Scheme 5). Herein, the treatment of methyl-terminated triethylene glycol monolayer-protected AuNPs (Me-EG_3_-AuNPs) with ω**-**carboxy tetraethylene glycol thiols (HOOC-EG_4_-SH) gave carboxy-functionalized AuNPs (HOOC-EG_4_-AuNPs). Peptide coupling of these HOOC-EG_4_-AuNPs with a DBCO-amine then yielded the DBCO-AuNPs. When these DBCO-AuNPs were treated with azide-decorated polymersomes (a class of artificial vesicles) [58], the AuNPs were successfully attached to the surface of the polymersomes (Scheme 6). Workentin and co-workers have also reported the successful use of SPAAC to synthesize peptide-decorated AuNPs [59] and nanomaterial hybrids containing single walled carbon nanotubes and AuNPs [60].

#### AuNP surface modification by CuAAC

**Scheme 7:**
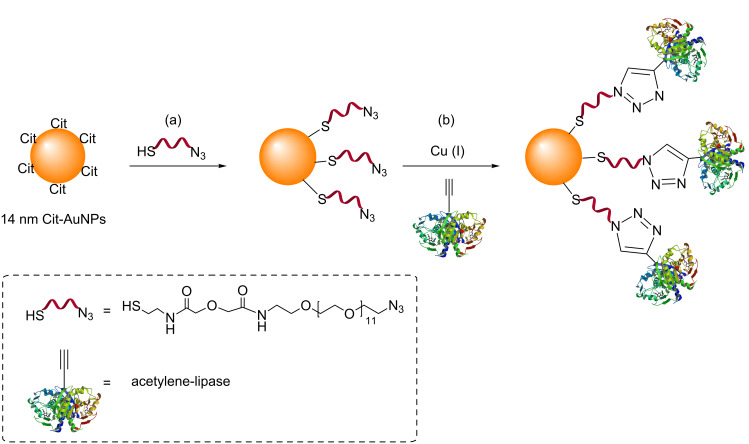
Functionalization of AuNPs with an azide containing thiol ligand, and subsequent attachment to an acetylene-functionalized lipase by CuAAC. Reagents and conditions: (a) H_2_O, rt, 18 h; (b) H_2_O, CuSO_4_, ascorbic acid, rt, 3 d. [47].

The distinct advantages of CuAAC over NCAAC, such as improved regioselectivity and rates of the reaction, motivated several groups to use CuAAC for the surface modification of AuNPs. In 2006, Brennan et al. demonstrated that enzyme–AuNP conjugates could be synthesized by CuAAC [47]. Azide-functionalized AuNPs were first synthesized by treating standard 14 nm Cit-AuNPs [28] with an a queous solution of an azide-containing thiol ligand (Scheme 7).

An acetylene-functionalized *Thermomyces lanuginosus* lipase was then attached to these azide-functionalized water-soluble AuNPs by CuAAC (Scheme 7). It was found that the enzyme retained its activity after the click reaction. However, the vast excesses of both Cu (a one million-fold excess relative to the azide) and lipase needed, the long reaction time (3 days), the extensive purification procedure required, and the poor overall conversion of azide to triazole (less than 1%) limited any further use of this procedure.

**Scheme 8:**
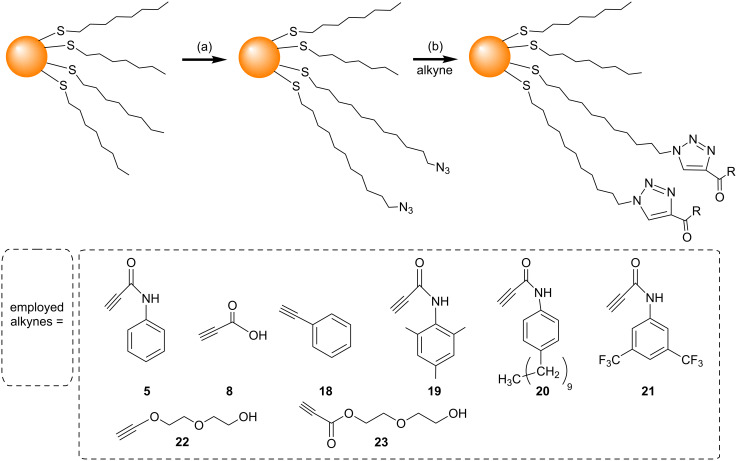
Surface modification of AuNPs using microwave-assisted CuAAC. Reagents and conditions: (a) HS(CH_2_)_11_N_3_, C_6_H_6_, rt, 7 h; (b) dioxane/*t-*BuOH/H_2_O or THF, CuSO_4_, sodium ascorbate, microwave heating (1000 W), 5–10 minutes [61].

In 2007, Sommer and Weck developed a simpler and more efficient method to perform CuAAC on the surface of AuNPs [61]. Herein microwave-assisted CuAAC was used to attach a variety of alkyne derivatives (**5**, **8**, and **18–23**) to azide-functionalized AuNPs (Scheme 8). The use of the microwave heating for the CuAAC reduced the reaction time to 5–10 minutes, and also gave almost quantitative conversion of the azides to triazoles. However, significant particle decomposition and/or aggregation were observed when the AuNPs were heated for more than 15 minutes in the microwave reactor.

**Scheme 9:**
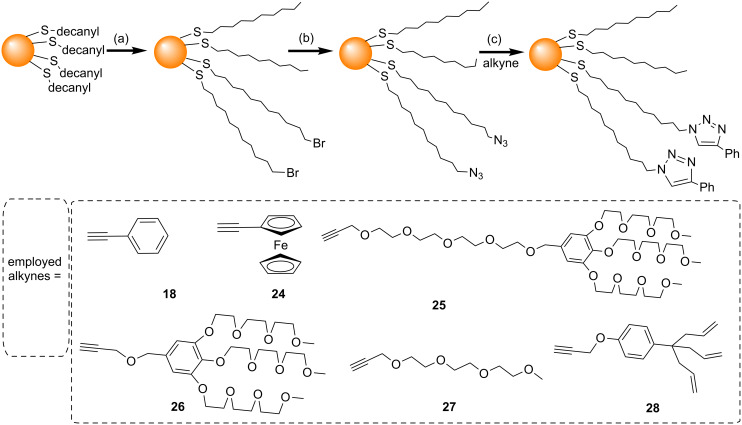
AuNP functionalization and efficient CuAAC with a range of alkynes reported by Boisselier et al. [62]. Reagents and conditions: (a) HS(CH_2_)_11_Br, DCM, rt, 5 d; (b) NaN_3_, DCM/DMSO, rt, 2 d; (c) CuSO_4_, sodium ascorbate, THF/H_2_O, 2 d, inert atmosphere.

Astruc and co-workers reported several modifications to try and increase the efficiency of CuAAC reactions of AuNPs [62]. They reasoned that one important consideration that needed to be addressed to enable an efficient click reaction was the solubility of the reagents; in particular alkanethiol-functionalized AuNPs are generally only soluble in organic solvents, whereas water is required to dissolve the CuSO_4_ catalyst. In order to circumvent this solubility problem, a homogenous water/THF solvent system was used, wherein a solution of the AuNPs in THF was added to either an aqueous solution containing water-soluble alkyne derivatives, or to a THF/water solution of organic soluble alkyne derivatives. The amount of ascorbic acid and Cu(I) was also increased to a stoichiometric amount with respect to the alkyne and azide. Finally the click reaction was performed under an inert atmosphere. The authors reported that if any of the above-mentioned conditions were not met, then the reaction gave a very poor yield of product. However, when all the conditions were fulfilled, the conversion of azide to triazole was virtually quantitative at room temperature. The reaction was performed with a variety of alkynes (**18** and **24–28**), and good results were obtained despite their variety of sizes and hydrophilicities (Scheme 9).

Astruc and co-workers have also reported that the use of copper(I) (hexabenzyl)tris(2-aminoethyl)amine bromide ([Cu(I)tren(CH_2_Ph_6_)]Br) instead of the CuSO_4_–ascorbic acid system improves the efficiency of CuAAC for the functionalization of AuNPs with a wide variety of organic, organometallic, polymeric and dendronic alkynes of different sizes and hydrophilicities [63–64]. CuAAC worked with a catalytic amount of [Cu(I)tren(CH_2_Ph_6_)]Br under ambient conditions with good yields and without any particle aggregation.

Following these reports, several groups have used the CuAAC reaction of AuNPs as a means for the detection of copper(II) salts [65–67] and ascorbic acid [68], and also for protein quantification (i.e., for proteins capable of reducing Cu(II) to Cu(I)) [69]. The basis of these detection systems was that two sets of AuNPs were synthesized, one of which was functionalized with azide-containing ligands and the other with alkyne-containing ligands. When these two were mixed in the presence of the required reagents and the corresponding analyte, a click reaction occurred causing aggregation of the AuNPs. The colour change and the surface plasmon resonance band shift induced by the particle aggregation thus served as the basis for the analyte detection.

### The functionalization of AuNPs with carbohydrates using AAC

#### The functionalization of AuNPs with carbohydrates using CuAAC

**Scheme 10:**
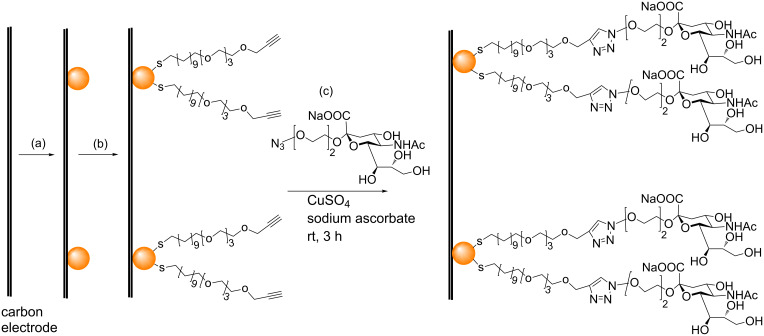
Schematic illustration of: (a) AuNP deposition on a carbon electrode; (b) formation of alkyne-terminated SAMs on these AuNPs; (c) conversion of these AuNPs into GAuNPs by CuAAC [74].

Although several groups have used the CuAAC to attach thiol-containing ligands to various sugars and then subsequently attach these sugar-containing thiol ligands to AuNPs [70–73], there has so far only been one study that reported the use of the CuAAC to click sugars directly onto the surface of AuNPs. In 2008, Chikae et al. reported the use of CuAAC to react alkyne-terminated thiol-functionalized AuNPs that had been deposited on a carbon electrode with an azide-terminated sialic acid derivative [74]. Firstly, AuNPs were electro-deposited on a carbon electrode. Then a solution of an alkyne-terminated disulphide (4,7,10,13,38,41,44,47-octaoxa-25,26-dithiapentaconta-1,49-diyne) was ‘dropped over’ the AuNP-electrode system to cover the AuNP surfaces with alkyne-terminated SAMs (Scheme 10). Next, a CuAAC reaction was used to couple the alkyne-functionalized AuNPs to an azide-linked sialic acid derivative, to produce GAuNPs attached to the carbon electrode. This sialic acid-functionalized GAuNP-carbon electrode system was then used for the detection of amyloid-β peptides [74], whose aggregation is responsible for Alzheimer’s disease [75].

In 2014, Fairbanks and co-workers reported a one-pot aqueous compatible method for making various triazole-linked glycoconjugates via intermediate glycosyl azides, which then underwent CuACC with a wide variety of alkynes [76]. The scarcity of reports on the use of the CuAAC for the functionalization of AuNPs with carbohydrates and the simplicity of the one-pot formation of glycosyl azides and their subsequent reaction with alkynes motivated us to investigate the use of this reaction sequence for the synthesis of GAuNPs.

**Scheme 11:**
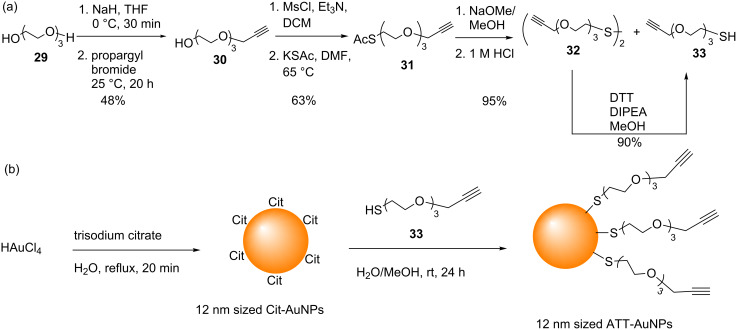
(a) Synthesis of the alkyne-terminated thiol (ATT) ligand **33**; (b) synthesis of 12 nm sized ATT-AuNPs by ligand exchange.

Firstly, the alkyne-terminated thiol (ATT) ligand **33** was synthesized as shown in Scheme 11a (see Supporting Information File 1 for full experimental data). Next, 12 nm ATT-AuNPs were synthesized by a ligand exchange reaction of 12 nm Cit-AuNPs (themselves synthesized by the Turkevich reaction) with the ATT **33** (Scheme 11b, see Supporting Information File 1 for full experimental data).

The particles obtained by this sequence were not soluble in either water or polar organic solvents, such as MeOH or MeCN, but they were soluble in non-polar solvents, such as DCM, CHCl_3_, and THF. The broad peaks corresponding to the ligand ATT **33** protons in the ^1^H NMR spectra of the purified ATT-AuNPs (Supporting Information File 1, Figure S1) confirmed the attachment of the ATT **33** to the AuNPs. Thermogravimetric analysis of ATT-AuNPs (Figure S2) and the size distribution of Cit-AuNPs and ATT-AuNPs (Figure S3) are also provided in Supporting Information File 1.

Whenever water-soluble ligands are used to perform exchange reactions on Cit-AuNPs, the wine-red colour of the AuNP solution (which corresponds to the dispersed state of the AuNPs as can be confirmed by TEM), and the SPR peak in the UV–vis spectrum are typically unchanged. However, in this case, when the water-insoluble ligand **33** was used, the solution turned purple (Supporting Information File 1, Figure S4), and the SPR peak shifted to a higher wavelength (523 nm to 541 nm) and became broader (Supporting Information File 1, Figure S5). Furthermore TEM revealed partial aggregation of the particles (Supporting Information File 1, Figure S6). However, despite this partial aggregation the ATT-AuNP solution was stable without any precipitation at least for three months when stored at 4 °C. Similar observations have been reported by Baranov et al. [77].

GlcNAc azide **34** was synthesized following the reported procedure (Supporting Information File 1) [76], and CuAAC of azide **34** and the AAT-AuNPs was attempted (Supporting Information File 1). Initially, only 1.5 mol % of CuSO_4_·5H_2_O (with respect to the ligands on the AAT-AuNPs) was used. However, ^1^H NMR analysis of the AuNPs revealed that the particles had not reacted with the glycosyl azide. Following the report of Boisselier et al. [62], a stoichiometric amount of CuSO_4_·5H_2_O was then used, and the reaction was performed under a nitrogen atmosphere. Firstly a solution of AAT-AuNPs in THF was added to an aqueous solution of the crude glycosyl azide, and then ascorbic acid, and finally a solution of CuSO_4_·5H_2_O dissolved in water were added. However, as soon as the CuSO_4_·5H_2_O was added, the particles precipitated; thus the click reaction failed and no GAuNPs were obtained. In further experiments the CuAAC was attempted using a solution of purified GlcNAc azide **34**. Water and THF were used as the solvent in a 1:1 ratio to be in line with the conditions reported by Boisselier et al. [62]. However, even with these conditions precipitation of the particles could not be prevented. Although this did confirm that neither the reagents nor byproducts from the azide synthesis were responsible for the particle aggregation, ultimately the reaction was unsuccessful. We include this finding in this comprehensive account in order to draw conclusions from it.

While several groups have demonstrated the successful use of CuAAC for the modification of AuNPs [47,61–62,78–79], at least three groups have reported that attempts to modify azide-functionalized AuNPs with alkyne derivatives by CuAAC either resulted in the reversible aggregation of the particles, or in negligible conversion [45,52,57]. For example, Fleming et al*.* reported attempts to increase the yield of the AAC using several different Cu-based catalyst systems [45]. As the particles (AuNPs functionalized with a mixture of decanethiol, Br-terminated undecanethiol, and azide-terminated undecanethiol) were insoluble in aqueous solutions, the most frequently used CuSO_4_-ascorbic acid system could not be used. Thus catalysts soluble in organic solvents, such as CuI, CuBr/2,6-lutidine, and bromotris(triphenylphosphinato)copper(I) were investigated. However in all cases, rapid and extensive particle aggregation or decomposition was observed. Limapichat et al. also reported similar results when Cu catalysts were used to accelerate the cycloaddition reaction [52]. In order to demonstrate the advantages of Cu-free SPAAC reactions, Workentin and co-workers compared Cu-free and Cu-catalysed click reactions with small water soluble AuNPs (particles functionalized with a mixture of Me-EG_3_-SH and N_3_-EG_4_-SH). Their attempts to perform CuAAC between the azide-modified AuNPs and alkynes (2-propyn-1-amine hydrochloride or 1-ethynylpyrene) in the presence of CuSO_4_ and sodium ascorbate resulted in particle decomposition [57]. However, when they performed SPAAC of the azide-modified AuNPs and dibenzocyclooctyne (DBCO)-amine, cycloaddition was complete after one hour, and gave the product in 60% yield. Hence, they suggested that the reaction of Cu(I) salts with the Au surface caused the particles to undergo aggregation/decomposition during the CuAAC [57]. It seems therefore that our attempts to synthesize GAuNPs using the one pot glycosyl azide/CuAAC reaction ran into the same limitations as reported by these three groups.

Boisselier et al. reported that by employing specific conditions, namely stoichiometric quantities of both CuSO_4_ and sodium ascorbate, a 1:1 mixture of water/THF as the reaction solvent, and a nitrogen atmosphere, CuAAC could be used to modify the surface of AuNPs [62]. However, it is notable that these reactions involved 2.5 nm AuNPs. Since the properties of AuNPs are highly dependent on their size, it may be that although the conditions reported by Boisselier et al. work well for smaller sized particles, however, may not be enough to overcome the precipitation of the larger sized AuNPs (>10 nm) caused by Cu as observed by some groups. Unfortunately our attempts to synthesize smaller sized (≈2 nm) ATT-AuNPs, either using two-phase (water/toluene) [49], or one-phase (MeOH) Brust–Schiffrin methods (BSM) [80] both resulted in the formation of decomposed/aggregated particles. We postulate that perhaps reaction of HAuCl_4_ with the terminal alkyne [81] of ATT **33** might have interfered with the Brust–Schiffrin reaction, and resulted in the formation of unstable AuNPs.

#### The functionalization of AuNPs with carbohydrates using SPAAC

**Scheme 12:**
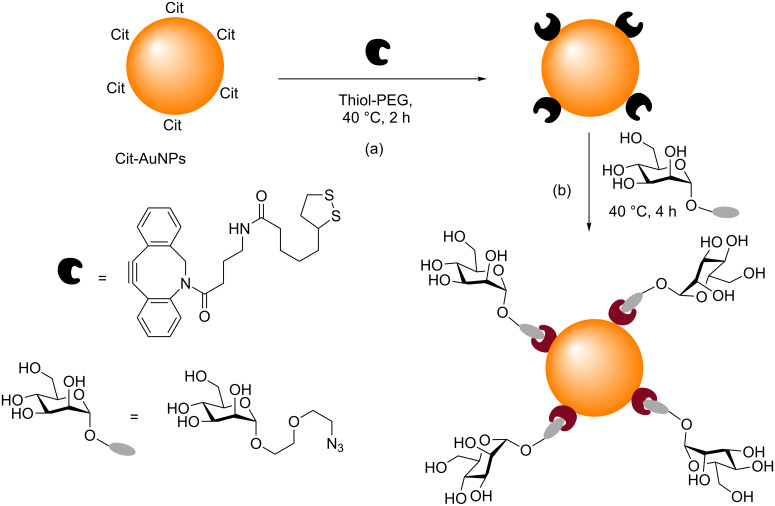
Synthesis of (a) cyclooctyne-functionalized AuNPs and (b) GAuNPs using SPAAC [82].

An alternative method for the functionalization of AuNPs with carbohydrates using click chemistry has recently been reported by Tian and co-workers [82]. They used SPAAC in their one-pot stepwise preparation of GAuNPs, and then used those particles as supramolecular glycoprobes for the rapid serological recognition of a cancer biomarker. Firstly, ligand exchange was performed on Cit-AuNPs by reaction with a THF solution of a cyclooctyne disulfide and an aqueous solution of tetraethylene glycol–thiol (dilutor ligands), to produce particles decorated with cyclooctynes (Scheme 12). These AuNPs then underwent SPAAC when an aqueous solution of a mannose-derived azide was added, to produce mannose-functionalized GAuNPs (Scheme 12). In the presence of the mannose-specific, dimeric lectin LcA (*Lens culinaris lectin*), these GAuNPs underwent aggregation. The GAuNP aggregates that were formed were then used as a supramolecular glycoprobe for the rapid detection of α-fetoprotein (AFP)-L3, a protein which binds strongly to LcA and is a serological biomarker for hepatocellular carcinoma (HCC). In this study AFP-L3 was captured on a microplate and the GAuNPs were added. The strong binding interaction between AFP-L3 and LcA caused disruption of the GAuNP-LcA aggregates, and a change in the optical density of the GAuNPs, which was measured with a microplate reader, enabling the detection of AFP-L3. Clearly this successful synthesis of GAuNPs by Tian and co-workers demonstrates that by employing SPAAC the Cu-induced aggregation/decomposition of AuNPs observed under CuAAC reactions as reported by some groups [45,52,57] can be avoided.

## Conclusion

With the combined features of an Au core and a surface decorated with multiple copies of biologically relevant carbohydrates, GAuNPs have become valuable tools in glycoscience. The simplicity and the versatility of the azide–alkyne Huisgen cycloaddition has stimulated several recent attempts to employ this type of reaction for the production of GAuNPs. When the non-catalysed azide–alkyne Huisgen cycloaddition was used for the surface modification of AuNPs, either the yields (i.e., the extent of the azide conversion to triazole) were poor, or long reaction times or hyperbaric conditions were required. There are somewhat conflicting reports in the literature with regard to the use of Cu(I)-catalysed azide–alkyne cycloaddition with AuNPs. Indeed although several groups have reported the successful use of CuAAC for the modification of AuNPs, both our own investigations, and those of number of other groups, have found that AuNP precipitation occurred under CuAAC reaction conditions [45,52,57]. Moreover the immediate precipitation of AuNPs that was observed upon the addition of CuSO_4_**^.^**5H_2_O implies that it was the Cu catalyst that caused precipitation. The precise reasons for this AuNP aggregation are not yet clear. Also, it seems difficult to extract a definite reason to explain as to why the CuAAC with AuNPs works for some groups while it fails in some other groups. However, in order to circumvent the limitations of CuAAC, SPAAC can be used as an alternative, and this provides a reliable method for the functionalization of AuNPs with carbohydrates using the azide–alkyne Huisgen cycloaddition.

## Supporting Information

File 1 Synthetic protocols and spectral and TEM characterisation for ATT **33** (Scheme 11), ATT-AuNPs (Scheme 11), GlcNAc azide **34**, and click reaction of ATT-AuNPs.

## References

[1] Thakor A S, Jokerst J, Zavaleta C, Massoud T F, Gambhir S S (2011). Nano Lett.

[2] Zhao P, Li N, Astruc D (2013). Coord Chem Rev.

[3] Elghanian R, Storhoff J J, Mucic R C, Letsinger R L, Mirkin C A (1997). Science.

[4] Saha K, Agasti S S, Kim C, Li X, Rotello V M (2012). Chem Rev.

[5] Turner M, Golovko V B, Vaughan O P H, Abdulkin P, Berenguer-Murcia A, Tikhov M S, Johnson B F G, Lambert R M (2008). Nature.

[6] Lee Y C, Lee R T (1995). Acc Chem Res.

[7] Jayaraman N (2009). Chem Soc Rev.

[8] de la Fuente J M, Barrientos A G, Rojas T C, Rojo J, Cañada J, Fernández A, Penadés S (2001). Angew Chem.

[9] Marradi M, Chiodo F, Garcia I, Penadés S (2013). Chem Soc Rev.

[10] Compostella F, Pitirollo O, Silvestri A, Polito L (2017). Beilstein J Org Chem.

[11] Marin M J, Schofield C L, Field R A, Russell D A (2015). Analyst.

[12] Zhao W, Brook M A, Li Y (2008). ChemBioChem.

[13] Schofield C L, Field R A, Russell D A (2007). Anal Chem.

[14] Otsuka H, Akiyama Y, Nagasaki Y, Kataoka K (2001). J Am Chem Soc.

[15] Richards S-J, Fullam E, Besra G S, Gibson M I (2014). J Mater Chem B.

[16] Niikura K, Nagakawa K, Ohtake N, Suzuki T, Matsuo Y, Sawa H, Ijiro K (2009). Bioconjugate Chem.

[17] Barrientos Á G, de la Fuente J M, Rojas T C, Fernández A, Penadés S (2003). Chem – Eur J.

[18] Lin C-C, Yeh Y-C, Yang C-Y, Chen G-F, Chen Y-C, Wu Y-C, Chen C-C (2003). Chem Commun.

[19] Lin C-C, Yeh Y-C, Yang C-Y, Chen C-L, Chen G-F, Chen C-C, Wu Y-C (2002). J Am Chem Soc.

[20] Svarovsky S A, Szekely Z, Barchi J J (2005). Tetrahedron: Asymmetry.

[21] Sundgren A, Barchi J J (2008). Carbohydr Res.

[22] de Paz J-L, Ojeda R, Barrientos Á G, Penadés S, Martín-Lomas M (2005). Tetrahedron: Asymmetry.

[23] Carvalho De Souza A, Halkes K M, Meeldijk J D, Verkleij A J, Vliegenthart J F G, Kamerling J P (2004). Eur J Org Chem.

[24] Carvalho de Souza A, Vliegenthart J F G, Kamerling J P (2008). Org Biomol Chem.

[25] Chien Y-Y, Jan M-D, Adak A K, Tzeng H-C, Lin Y-P, Chen Y-J, Wang K-T, Chen C-T, Chen C-C, Lin C-C (2008). ChemBioChem.

[26] Marradi M, Martín-Lomas M, Penadés S (2010). Adv Carbohydr Chem Biochem.

[27] Schofield C L, Mukhopadhyay B, Hardy S M, McDonnell M B, Field R A, Russell D A (2008). Analyst.

[28] Frens G (1973). Nature (London), Phys Sci.

[29] Poonthiyil V, Golovko V B, Fairbanks A J (2015). Org Biomol Chem.

[30] Poonthiyil V, Nagesh P T, Husain M, Golovko V B, Fairbanks A J (2015). ChemistryOpen.

[31] Chen F, Li X, Hihath J, Huang Z, Tao N (2006). J Am Chem Soc.

[32] Halkes K M, Carvalho De Souza A, Maljaars C E P, Gerwig G J, Kamerling J P (2005). Eur J Org Chem.

[33] Nagahori N, Abe M, Nishimura S-I (2009). Biochemistry.

[34] Telli F C, Demir B, Barlas F B, Guler E, Timur S, Salman Y (2016). RSC Adv.

[35] Wang X, Ramström O, Yan M (2010). Anal Chem.

[36] Wang X, Ramström O, Yan M (2009). J Mater Chem.

[37] Kolb H C, Finn M G, Sharpless K B (2001). Angew Chem, Int Ed.

[38] Huisgen R (1963). Angew Chem, Int Ed Engl.

[39] Liang L, Astruc D (2011). Coord Chem Rev.

[40] Tornøe C W, Christensen C, Meldal M (2002). J Org Chem.

[41] Himo F, Lovell T, Hilgraf R, Rostovtsev V V, Noodleman L, Sharpless K B, Fokin V V (2005). J Am Chem Soc.

[42] Meldal M, Tornøe C W (2008). Chem Rev.

[43] Kolb H C, Sharpless K B (2003). Drug Discovery Today.

[44] Rostovtsev V V, Green L G, Fokin V V, Sharpless K B (2002). Angew Chem.

[45] Fleming D A, Thode C J, Williams M E (2006). Chem Mater.

[46] Li N, Binder W H (2011). J Mater Chem.

[47] Brennan J L, Hatzakis N S, Tshikhudo T R, Dirvianskyte N, Razumas V, Patkar S, Vind J, Svendsen A, Nolte R J M, Rowan A E (2006). Bioconjugate Chem.

[48] Ismaili H, Alizadeh A, Snell K E, Workentin M S (2009). Can J Chem.

[49] Brust M, Walker M, Bethell D, Schiffrin D J, Whyman R (1994). J Chem Soc, Chem Commun.

[50] Collman J P, Devaraj N K, Chidsey C E D (2004). Langmuir.

[51] Thode C J, Williams M E (2008). J Colloid Interface Sci.

[52] Limapichat W, Basu A (2008). J Colloid Interface Sci.

[53] Jewett J C, Bertozzi C R (2010). Chem Soc Rev.

[54] Debets M F, van Berkel S S, Dommerholt J, Dirks A J, Rutjes F P J T, van Delft F L (2011). Acc Chem Res.

[55] Evans H L, Slade R L, Carroll L, Smith G, Nguyen Q-D, Iddon L, Kamaly N, Stöckmann H, Leeper F J, Aboagye E O (2012). Chem Commun.

[56] Ning X, Guo J, Wolfert M A, Boons G-J (2008). Angew Chem, Int Ed.

[57] Gobbo P, Mossman Z, Nazemi A, Niaux A, Biesinger M C, Gillies E R, Workentin M S (2014). J Mater Chem B.

[58] Amos R C, Nazemi A, Bonduelle C V, Gillies E R (2012). Soft Matter.

[59] Wang X, Gobbo P, Suchy M, Workentin M S, Hudson R H E (2014). RSC Adv.

[60] Gobbo P, Novoa S, Biesinger M C, Workentin M S (2013). Chem Commun.

[61] Sommer W J, Weck M (2007). Langmuir.

[62] Boisselier E, Salmon L, Ruiz J, Astruc D (2008). Chem Commun.

[63] Li N, Zhao P, Salmon L, Ruiz J, Zabawa M, Hosmane N S, Astruc D (2013). Inorg Chem.

[64] Zhao P, Grillaud M, Salmon L, Ruiz J, Astruc D (2012). Adv Synth Catal.

[65] Zhou Y, Wang S, Zhang K, Jiang X (2008). Angew Chem.

[66] Hua C, Zhang W H, De Almeida S R M, Ciampi S, Gloria D, Liu G, Harper J B, Gooding J J (2012). Analyst.

[67] Zhang Z, Li W, Zhao Q, Cheng M, Xu L, Fang X (2014). Biosens Bioelectron.

[68] Zhang Y, Li B, Xu C (2010). Analyst.

[69] Zhu K, Zhang Y, He S, Chen W, Shen J, Wang Z, Jiang X (2012). Anal Chem.

[70] Papp I, Sieben C, Ludwig K, Roskamp M, Böttcher C, Schlecht S, Herrmann A, Haag R (2010). Small.

[71] Marín M J, Rashid A, Rejzek M, Fairhurst S A, Wharton S A, Martin S R, McCauley J W, Wileman T, Field R A, Russell D A (2013). Org Biomol Chem.

[72] Wei J, Zheng L, Lv X, Bi Y, Chen W, Zhang W, Shi Y, Zhao L, Sun X, Wang F (2014). ACS Nano.

[73] Martos-Maldonado M C, Thygesen M B, Jensen K J, Vargas-Berenguel A (2013). Eur J Org Chem.

[74] Chikae M, Fukuda T, Kerman K, Idegami K, Miura Y, Tamiya E (2008). Bioelectrochemistry.

[75] Miura Y, Yasuda K, Yamamoto K, Koike M, Nishida Y, Kobayashi K (2007). Biomacromolecules.

[76] Lim D, Brimble M A, Kowalczyk R, Watson A J A, Fairbanks A J (2014). Angew Chem.

[77] Baranov D, Kadnikova E N (2011). J Mater Chem.

[78] Zhang M-X, Huang B-H, Sun X-Y, Pang D-W (2010). Langmuir.

[79] Kim Y-P, Daniel W L, Xia Z, Xie H, Mirkin C A, Rao J (2010). Chem Commun.

[80] Brust M, Fink J, Bethell D, Schiffrin D, Kiely C (1995). J Chem Soc, Chem Commun.

[81] Hashmi A S K (2007). Chem Rev.

[82] He X-P, Hu X-L, Jin H-Y, Gan J, Zhu H, Li J, Long Y-T, Tian H (2015). Anal Chem.

