# Supplementary material for: Recent applications of click chemistry for the functionalization of gold nanoparticles and their conversion to glyco-gold nanoparticles
Source: Beilstein J Org Chem. 2018 Jan 3;14:11–24. doi: 10.3762/bjoc.14.2 (PMC5769080; doi:10.3762/bjoc.14.2)
Supplement: File 1 — Synthetic protocols and spectral and TEM characterisation for ATT 33 (Scheme 11), ATT-AuNPs (Scheme 11), GlcNAc azide 34, and click reaction of ATT-AuNPs. [file Beilstein_J_Org_Chem-14-11-s001.pdf]

Supporting Information
for
Recent applications of click chemistry for the functionalization of gold nanoparticles and their conversion to glyco-gold nanoparticles

Vivek Poonthiyil*[1], Thisbe K. Lindhorst[1], Vladimir B. Golovko[2,3] and Antony J. Fairbanks*[2,4]

Address: [1] Otto Diels Institute of Organic Chemistry, Christiana Albertina University of Kiel, Otto-Hahn-Platz 3/4, Kiel, 24098, Germany, [2]Department of Chemistry, University of Canterbury, Private Bag 4800, Christchurch, 8140, New Zealand, [3]The MacDiarmid Institute for Advanced Materials and Nanotechnology, Wellington, 6140, New Zealand and [4] Biomolecular Interaction Centre, University of Canterbury, Private Bag 4800, Christchurch 8140, New Zealand

Email: Antony J. Fairbanks - antony.fairbanks@canterbury.ac.nz, Vivek Poonthiyil - vpoonthiyil@oc.uni-kiel.de

*Corresponding author

Synthetic protocols and spectral and TEM characterisation for ATT 33 (Scheme 11), ATT-AuNPs (Scheme 11), GlcNAc azide 34, and click reaction of ATT-AuNPs

All chemical reagents were analytical grade and were used as supplied without further purification, unless otherwise stated. Solvents were removed under reduced pressure using a Buchi™ rotary evaporator. Thin layer chromatography (tlc) was carried out on Merck Silica Gel $60F_{254}$ aluminium backed plates. Plate visualisation was achieved using a UV lamp (λ_{max} = 254 or 365 nm), and/or ammonium molybdate (5% in 2M H_2SO_4). Flash column chromatography was carried out using Sorbsil C60 40/60 silica. Proton (δ_H) and carbon (δ_C) nuclear magnetic resonance spectra were recorded on Bruker AV400 (400 MHz), and Bruker AV500 (500 MHz) spectrometers. All chemical shifts are quoted on the δ-scale in ppm using residual solvent as an internal standard. High resolution mass spectra were recorded a Bruker FT-ICR mass spectrometer using electrospray ionisation (ESI) or chemical ionisation (CI) techniques as stated. The *m*/*z* values are reported in Daltons. TEM images of AuNPs were obtained using a Philips CM200 TEM operating at 200 kV. Samples for TEM imaging were prepared by dropping 2 µL of a freshly prepared solution of nanostructured Au onto a holey

carbon-coated copper grid (300 mesh, SPI Supplies, USA) and drying at room temperature. UV–vis absorption spectra were recorded using a Varian Cary 100 UV–vis spectrophotometer. Thermogravimetric analysis (TGA) was performed with an Alphatech SDT Q600 TGA/DSC apparatus, on 6–8 mg of purified, dry materials (alumina crucible was used as the sample holder), under N_2 (with a flow rate of 100 mL/min), recording data from 25 to 1000 °C at a heating rate of 10 °C/min. Infrared spectra were recorded on a Perkin-Elmer Spectrum One.

3,6,9-Trioxadodec-11-yne-1-ol (30) [1]

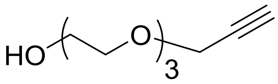

Triethylene glycol **29** (15.02 g, 100 mmol) was dissolved in 40 mL of dry THF and stirred under nitrogen at 0 ^{o}C. NaH (2.60 g, 65 mmol, 60% dispersion in mineral oil) was added slowly, and the mixture stirred for 30 min. Propargyl bromide (7.9 mL, 50 mmol) was then added dropwise, and the mixture was stirred at 0 ^{o}C for a further 2 h, and then at 25 ^{o}C for 20 h. After this time, tlc (petrol/EtOAc, 1:3) showed the complete consumption of the starting material (R_f 0) and the formation of a major product (R_f 0.3). The reaction was quenched by the careful addition of MeOH (20 mL), and the mixture was then filtered through Celite® and concentrated in vacuo. The residue was purified by flash column chromatography (petrol/EtOAc, 2:1) to give 3,6,9-trioxadodec-11-yne-1-ol (**30**, 9 g, 48%) as a yellow oil. δ_H (400 MHz, $CDCl_3$)[1] 2.26 (1H, br s, OH), 2.42 (1H, t, *J* 2.3 Hz, $H_2CCC\underline{H}$), 3.55 - 3.79 (12H, m, 6 x CH_2O), 4.20 (2H, d, *J* 2.3 Hz, $\underline{H_2}CCCH$); HRMS (ESI) Calcd. For $C_9H_{17}O_4$ (MH^+) 189.1049. Found 189.1054.

1-Acetylthio-3,6,9-trioxadodec-11-yne (31) [2]

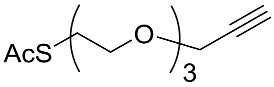

Alkyne **30** (9 g, 47.8 mmol) was dissolved in dry DCM (150 mL) and stirred under nitrogen at 0 °C. Mesyl chloride (7.4 mL, 96 mmol) was added dropwise, followed by triethylamine (13.3 mL, 96 mmol). The reaction mixture was then warmed to rt. After 2h, tlc (petrol:EtOAc, 2:1) indicated the formation of a single product (R_f 0.4), and the complete consumption of the starting material (R_f 0.2). The reaction was quenched by the addition of MeOH (30 mL) and concentrated in vacuo. The residue was dissolved in DMF (150 mL), potassium thioacetate (22 g, 191 mmol) was added, and the reaction was then stirred at 65 °C. After 16 h, tlc (petrol/EtOAc, 2:1) indicated the formation of a major product (R_f 0.6), and the complete consumption of the starting material (R_f 0.4). The reaction mixture was cooled to rt, diluted with EtOAc (100 mL), and washed successively with an aqueous solution of saturated $NaHCO_3$ (2 × 60 mL), H_2O (2 × 60 mL), and brine (30 mL). The organic layer was

separated, dried (Na_2SO_4), filtered, and concentrated in vacuo. The residue was purified by flash chromatography (petrol:EtOAc, 10:1) to afford 1-acetylthio-3,6,9-trioxadodec-11-yne (**31**, 7.4 g, 63%) as a yellow syrup. ν_{max} (KBr disc) 3271 (s, $H_2CC\underline{C\text{-}H}$) 2865 (s, $H_2C\underline{C\text{-}H}$), 1685 (s, C=O) cm^{-1}. δ_H (400 MHz, $CDCl_3$) [2] 2.31 (1H, s, CH_3CO), 2.42 (1H, t, *J* 2.3 Hz, $H_2CCC\underline{H}$), 3.08 (2H, t, *J* 6.5 Hz, CH_2S), 3.53 - 3.73 (10H, m, 5 x CH_2O), 4.20 (2H, d, *J* 2.3 Hz, $\underline{H_2}CCCH$); HRMS (ESI) Calcd. For $C_{11}H_{19}O_4S$ (MH^+) 247.0999. Found 247.0990.

1,1`-Dithiobis(3,6,9-trioxadodec-11-yne) (32)

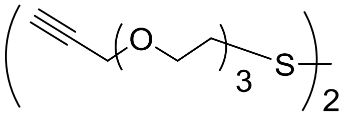

NaOMe (an 8.1 M solution in MeOH) was added to a solution of compound **31** (2.0 g, 8.1 mmol) in MeOH (50 mL), and the solution was stirred at rt under nitrogen. After 2 h, the solution was neutralized (confirmed by pH paper) by the addition of 1 M aqueous HCl, and then air-oxidized by bubbling air continuously through the solution for a total of 72 h. The solvent was then removed in vacuo to afford disulfide **32** (1.6 g, 95%) as a yellow syrup; ν_{max} (KBr disc) 3253 (w, $H_2CC\underline{C\text{-}H}$), 2865 (s, $H_2C\underline{C\text{-}H}$ stretch), 1349 (w, $H_2C\underline{C\text{-}H}$ bend), 1092 (s, C-O) cm^{-1}; δ_H (400 MHz, $CDCl_3$) 2.42 (1H, t, *J* 2.2 Hz, $H_2CCC\underline{H}$), 2.86 (2H, t, *J* 8 Hz, CH_2S), 3.55 - 3.77 (10H, m, 5 x CH_2O), 4.18 (2H, d, *J* 2.4 Hz, $\underline{H_2}CCCH$); δ_C (100 MHz, $CDCl_3$) 38.4 (t, CH_2S), 58.4 (t, $H_2\underline{C}CCH$), 69.1 (t, CH_2O), 69.6 (t, CH_2O), 70.3 (t, CH_2O), 70.4 (t, CH_2O), 70.5 (t, CH_2O), 74.5 (d, $H_2CC\underline{C}H$), 79.6 (s, $H_2C\underline{C}CH$); HRMS (ESI) Calcd. For $C_{18}H_{30}O_6S_2Na$ (MNa^+) 429.1376. Found 429.1375

3,6,9-Trioxadodec-11-yne-1-thiol (ATT, 33)

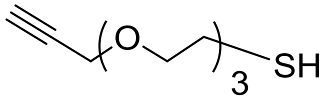

Dithiothreitol (DTT) (569 mg, 3.69 mmol) was added to a solution of the disulfide **32** (300 mg, 0.739 mmol) and *N,N*-diisopropylethylamine (DIPEA, 0.65 mL, 3.69 mmol) in MeOH and stirred under nitrogen at rt. After 3 h, tlc (petrol/EtOAc, 1:1) indicated the formation of a single product (R_f 0.4), and the complete consumption of the starting material (R_f 0.3). The reaction mixture was concentrated in vacuo, diluted with EtOAc (30 mL) and washed successively with 1 M aqueous HCl solution (2 x 30 mL), a saturated aqueous solution of $NaHCO_3$ (2 × 30 mL), H_2O (2 × 30 mL), and brine (30 mL). The organic layer was separated, dried (Na_2SO_4), filtered, and concentrated in vacuo. The residue was purified by flash chromatography (petrol/EtOAc, 10:1) to afford 3,6,9-trioxadodec-11-yne-1-thiol (**33**, (270 mg, 90%) as a yellow syrup. ν_{max} (KBr disc) 3250 (w, H_2CCC-H), 2865 (s, H_2CC-H), 1348 (w, H_2CC-H bend), 1090 (s, C-O) cm^{-1}; δ_H (400 MHz, $CDCl_3$) 1.56 (1H, t, *J* 8 Hz, SH), 2.42 (1H, t, *J* 2.2 Hz, H_2CCCH), 2.66 (2H, m, CH_2S), 3.55 - 3.71 (10H, m, 5 x CH_2O), 4.18 (2H, d, *J* 2.4 Hz, H_2CCCH); δ_C (100 MHz, $CDCl_3$) 23.5 (t, CH_2S), 58.4 (t, H_2CCCH), 69.1 (t, CH_2O), 70.2 (t, CH_2O), 70.4 (t, CH_2O), 70.5 (t, CH_2O), 72.8 (t, CH_2O), 74.5 (d, H_2CCCH), 79.6 (s, H_2CCCH); HRMS (ESI) Calcd. For $C_9H_{16}O_3SNa$ (MNa^+) 227.0712. Found 227.0721.

2-Acetamido-2-deoxy-β-D-glucopyranosyl azide (34) [3]

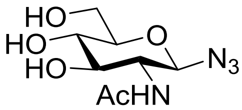

N-Acetyl-D-glucosamine (250 mg, 1.13 mmol) and triethylamine (0.8 mL, 5.7 mmol) were stirred in D_2O/MeCN (4:1, 5 mL) and cooled to 0 °C. 2-Azido-1,3-dimethylimidazolinium hexafluorophosphate (ADMP) (0.95 g, 3.4 mmol) was added. After 1 h, tlc ($CHCl_3$/MeOH, 2:1) indicated complete consumption of starting material (R_f 0.2) and the formation of two major products (R_f 0.5, 0.6). The reaction mixture was acidified to pH 2 by dropwise addition of aqueous 1.2 M HCl, and then

neutralized by addition of $NaHCO_3$ (5 mL of a saturated aqueous solution). The solution pH was followed by using pH paper. The reaction mixture was concentrated, and the residue was dissolved in ethanol (10 mL), filtered through Celite®, and concentrated in vacuo. The residue was then redissolved in water (10 mL), washed with DCM (2 × 20 mL), filtered through a column of Amberlite® IR120 (H^+, previously treated with aqueous 1 M NaOH solution), and concentrated in vacuo. Purification by flash column chromatography ($CHCl_3$/MeOH, 5:1) gave 2-acetamido-2-deoxy-β-D-glucopyranosyl azide (**34**, 240 mg, 84%) as a white solid; m.p. 127-131 °C [lit. m.p. 125-130 °C][3]; $[\alpha]_D^{20}$ -34 (*c*, 1.0 in MeOH) [lit. $[\alpha]_D^{20}$ −34 (*c*, 1.0 in MeOH)][3]; δ_H (400 MHz, D_2O)[3]; HRMS (ESI) Calcd. For $C_8H_{14}N_4O_5$ (MNa^+) 269.0862. Found 269.0852.

3,6,9-Trioxadodec-11-yne-1-thiol-functionalized AuNPs (ATT-AuNPs)

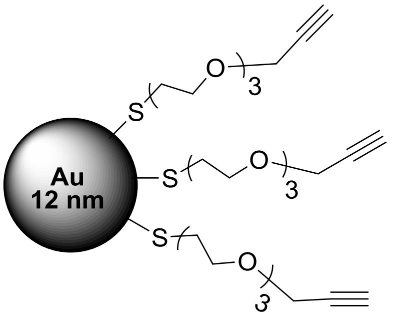

An aqueous solution of trisodium citrate (10 mL, 42.9 mM) was added to a boiling aqueous solution of $HAuCl_4{\cdot}3H_2O$ (100 mL, 1 mM) with constant stirring (500 rpm). After 15 min, the reaction mixture was cooled to rt, and a solution of ATT **33** (150 mg, 0.74 mmol) in MeOH (50 mL) was added. The reaction was stirred for 24 h at rt. The reaction mixture was then concentrated in vacuo, and the residue was washed with water (3 × 10 mL). The crude product was then dissolved in MeOH and purified by centrifugation (4 × 6000 rpm) to afford ATT-AuNPs (20 mg). TEM: 12.0 ± 1.4 nm; IR (KBr): 2921 (s, $H_2C\underline{C-H}$ stretch), 1458 (w, $H_2C\underline{C-H}$ bend), 1084 (s, C-O) cm^{-1}; UV: 541 nm; TGA: ligand 45.4%, Au 54.6%; δ_H (400 MHz, $CDCl_3$) 2.44 (br. s, $H_2CCC\underline{H}$), 2.70-3.03 (br. m, CH_2S), 3.50 - 3.80 (br. m, CH_2O), 4.20 (br. s, $\underline{H_2}CCCH$); HRMS (ESI) Calcd. For $C_9H_{16}O_3SNa$ (MNa^+) 227.0712. Found 227.0721.

Attempted click reaction on the surface of ATT-AuNPs

N-Acetyl-D-glucosamine **34** (10 mg, 0.045 mmol) and triethylamine (32 µL, 0.26 mmol) were dissolved in D_2O/MeCN (4:1, 0.2 mL) and the mixture was cooled to 0 °C. ADMP (38 mg, 0.13 mmol) was then added, and the reaction was stirred at 0 °C. After 1 h, tlc ($CHCl_3$/MeOH, 2:1) indicated the complete consumption of the starting material (R_f 0.2), and the formation of two major products (R_f 0.5, 0.6). The reaction mixture was acidified to pH 2 by the dropwise addition of aqueous 1.2 M HCl and then neutralized by addition of saturated aqueous $NaHCO_3$ solution. The solution pH was followed by using pH paper. ATT-AuNP (3 mg) dissolved in 2 mL of THF, L-ascorbic acid (25 mg, 0.14 mmol), $CuSO_4{\cdot}5H_2O$ (2.5 mg, 0.01 mmol) dissolved in H_2O were added to the reaction and stirred under nitrogen at rt. Aggregated/decomposed particles were obtained.

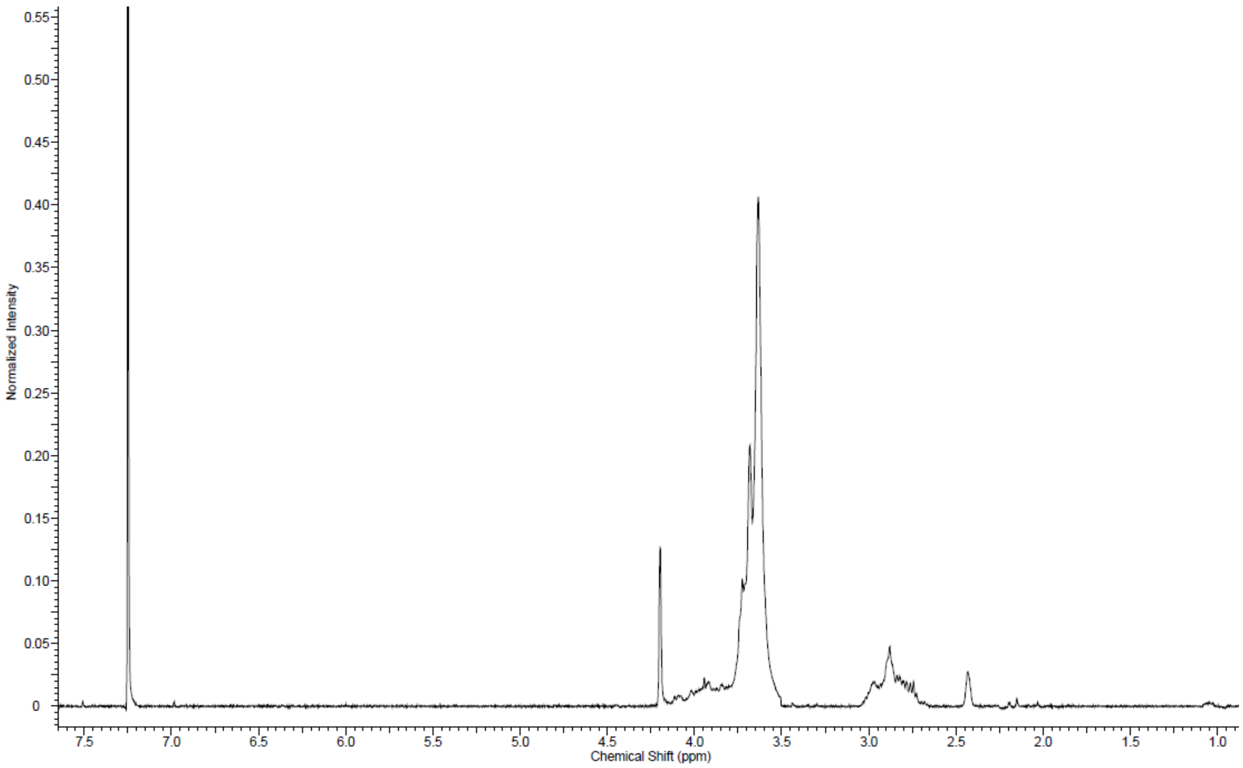

Figure S1: ^{1}H NMR spectrum of ATT-AuNPs.

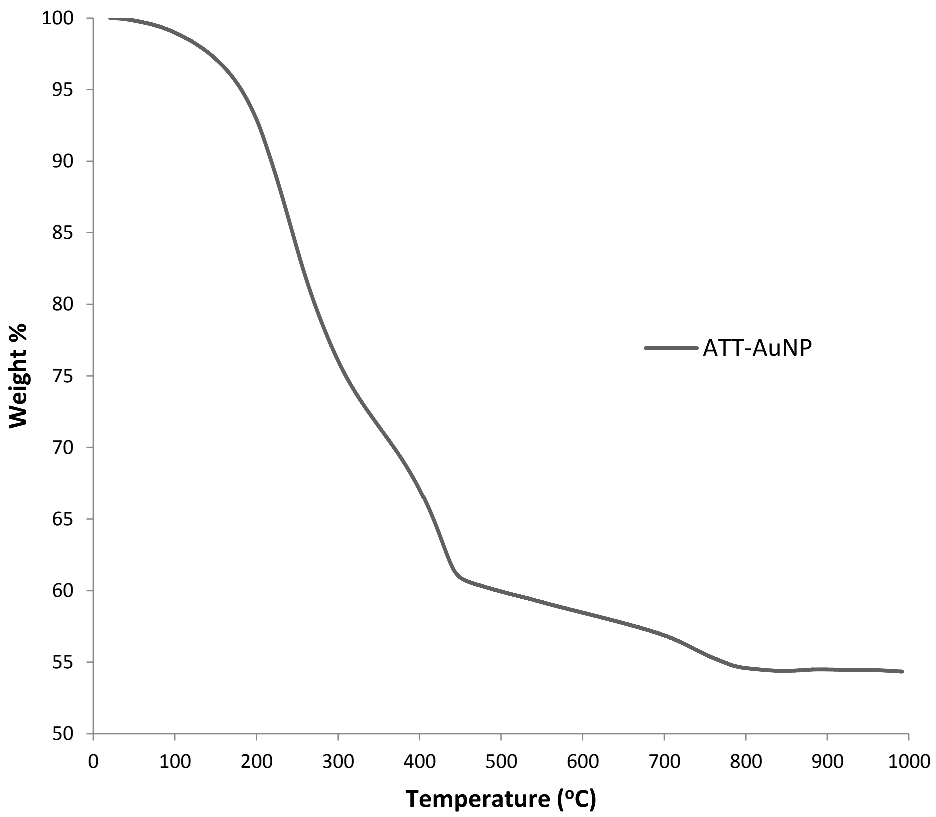

Figure S2: TGA plot of ATT-AuNPs.

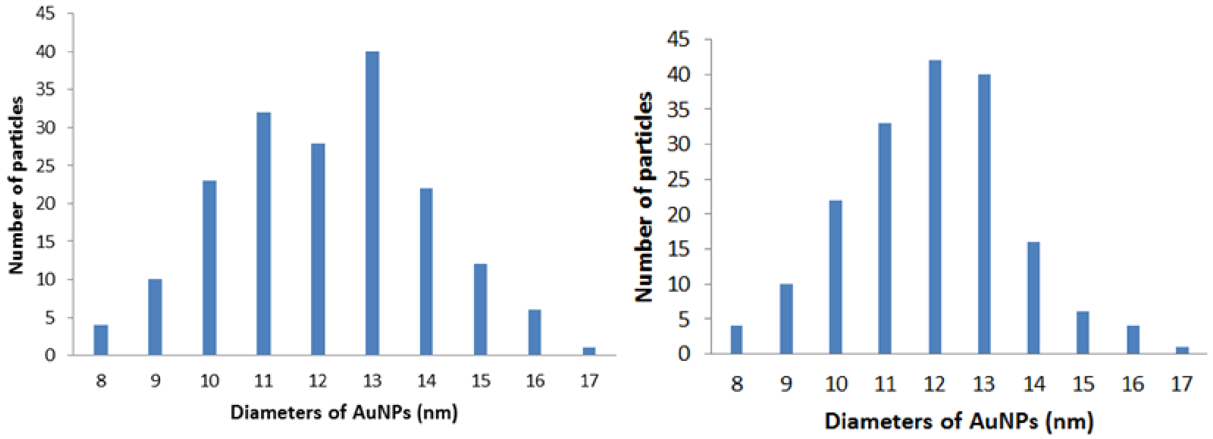

Figure S3: Size distribution histograms of Cit-AuNPs (12.1 ± 1.5 nm) and ATT-AuNPs (12.0 ± 1.4 nm).

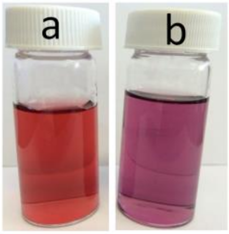

Figure S4: Photographs of AuNP solutions (a) Cit-AuNPs in water and (b) ATT-AuNPs in DCM.

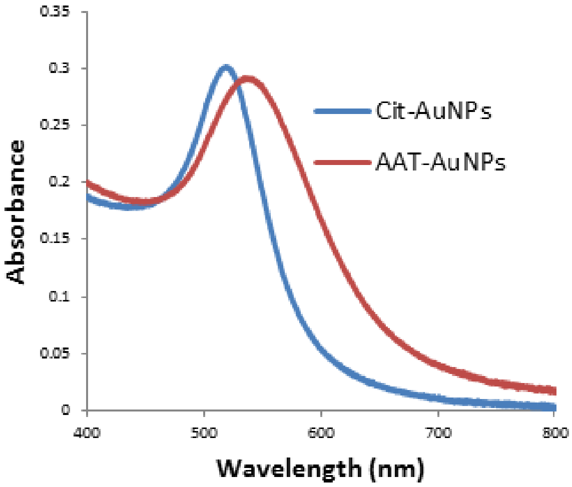

Figure S5: UV–vis absorbance spectra of the Cit-AuNPs in water and AAT-AuNPs in DCM.

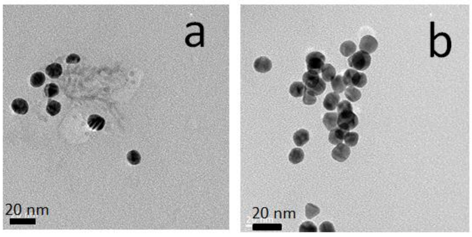

Figure S6: Representative TEM images of (a) Cit-AuNPs and (b) AAT-AuNPs.

References

1. Lu, G.; Lam, S.; Burgess, K. *Chem. Commun.* **2006**, 1652-1654.
2. Ning, X.; Lee, S.; Wang, Z.; Kim, D.; Stubblefield, B.; Gilbert, E.; Murthy, N. *Nat. Mater.* **2011**, *10*, 602-607.
3. Lim, D.; Brimble, M. A.; Kowalczyk, R.; Watson, A. J. A.; Fairbanks, A. J. *Angew. Chem.* **2014**, *126*, 12101-12105.
